# Quasispecies theory in the context of population genetics

**DOI:** 10.1186/1471-2148-5-44

**Published:** 2005-08-17

**Authors:** Claus O Wilke

**Affiliations:** 1 Keck Graduate Institute of Applied Life Sciences, 535 WatsonDrive, Claremont, California 91711, USA; 2 Digital Life Laboratory, California Institute of Technology, Mail Code 136-93, Pasadena, California 91125, USA

## Abstract

**Background:**

A number of recent papers have cast doubt on the applicability of the quasispecies concept to virus evolution, and have argued that population genetics is a more appropriate framework to describe virus evolution than quasispecies theory.

**Results:**

I review the pertinent literature, and demonstrate for a number of cases that the quasispecies concept is equivalent to the concept of mutation-selection balance developed in population genetics, and that there is no disagreement between the population genetics of haploid, asexually-replicating organisms and quasispecies theory.

**Conclusion:**

Since quasispecies theory and mutation-selection balance are two sides of the same medal, the discussion about which is more appropriate to describe virus evolution is moot. In future work on virus evolution, we would do good to focus on the important questions, such as whether we can develop accurate, quantitative models of virus evolution, and to leave aside discussions about the relative merits of perfectly equivalent concepts.

## Background

Quasispecies theory describes the evolution of an infinite population of asexual replicators at high mutation rate [1,2]. Quasispecies theory is often cited as the theory to describe the evolution of RNA viruses [3], but in recent years several authors have questioned whether quasispecies theory has any relevance for virus evolution [4-7]. Esteban Domingo has responded to this criticism from an experimentalist's point of view [8]. However, the fundamental issue in this discussion is of theoretical nature, and has not yet been addressed in detail. The fundamental issue is whether quasispecies theory and population genetics are two competing theories, and whether virology ulitmately has to decide for or against one or the other. Some quasispecies opponents have argued that quasispecies theory contradicts population genetics (e.g. "This model contrasts sharply with conventional population genetics models ..." in [7]), and that there is no evidence that favors quasispecies theory over classical population genetics [6]. On the other hand, some quasispecies proponents have also voiced the position that quasispecies theory goes beyond population genetics, and virologists in general have frequently used the term *quasispecies *inappropriately (see e.g. the discussion on this topic by Eigen, Ref. [9]). I find this discussion somewhat frustrating, because quasispecies theory is simply a subset of theoretical population genetics, and it is mathematically equivalent to the theory of mutation-selection balance. The only real difference between quasispecies theory and mutation-selection balance is that they have been developed largely independently by two separate schools of research, and that these schools of research have often focused on somewhat different questions and special cases. Quasispecies theory treats multiple loci, whereas early work on mutation-selection balance has focused on one- or two-locus models. On the other hand, most work on population genetics considers finite populations and includes stochastic effects, whereas quasispecies theory is first and foremost a deterministic description of infinite populations.

Quasispecies theory has its origin in a seminal paper written by Eigen in 1971 [10], in which he studied the error-prone self-replication of biological macromolecules, primarily with the goal of understanding the origin of life. However, Eigen did not yet use the term *quasispecies *in this 1971 paper; he coined this term in a later paper coauthored with Peter Schuster [11]. These early papers by Eigen, Schuster, and coworkers (reviewed in [1,2]) were some of the first to study theoretically the extreme nucleotide heterogeneity caused by highly error-prone replication. As a consequence, they generated interest among researchers working on RNA viruses, as these viruses were found to replicate at high mutation rates and have extremely polymorphic populations [3,12-14].

Eigen's papers also generated substantial interest among theoreticians (mostly physicists), who found the description of highly error-prone replication an interesting theoretical challenge. Unfortunately, much of the theoretical follow-up work [15-21] has focused on a particular fitness landscape, the single-peak (or master sequence) fitness landscape, in which a single sequence (the master sequence) has superior fitness 1 + *s*, and all other sequences have inferior fitness 1. As a result, much of the generality of Eigen's original work, as well as its connection to population genetics, have been obscured, and the conclusions of these special-case studies are frequently taken to be general predictions of quasispecies theory.

Because of the development of quasispecies theory independently from population genetics, and because of the widespread emphasis on a single fitness landscape in quasispecies theory, many authors now hold a set of beliefs about quasispecies theory that do not correspond to the actual predictions of the theory. These beliefs are:

1. Quasispecies theory is at odds with population genetics.

2. Quasispecies theory is inapplicable if populations are finite and there is neutral drift.

3. Quasispecies theory predicts an error threshold.

In the next three sections, I will address each of these points in detail. However, first I have to define what exactly I mean by quasispecies theory.

Throughout this paper, by quasispecies theory I mean specifically Eq. (6) in Ref. [11],



where *x*_*i*_(*t*) is the concentration of sequence *i*, *W*_*ij *_= *A*_*j*_*Q*_*ij *_is the product of the replication rate (fitness) *A*_*j *_of sequence *j *and the mutation probability *Q*_*ij *_from sequence *j *to *i*, and *E*(*t*) is the total production of new sequences,



In my definition of quasispecies theory, I also include straightforward generalizations of the above equation that have been used in the quasispecies literature, such as the discrete-time quasispecies equation, which can be written as [22,23]



and leads to the same steady-state solution as Eq. (1). Both Eqs. (1) and (3) can be mapped onto linear equations, and then solved by diagonalizing the matrix *W*_*ij*_. In both cases, the steady-state solution is given by the dominant eigenvector of *W*_*ij*_.

The mapping onto a linear system assumes that the *W*_*ij*_, which consist of the fitness landscape (as given by the *A*_*j*_) and the mutation landscape (as given by the *Q*_*ij*_), are constants. In the most general case, fitness will depend on the mutant frequencies *x*_*i*_, as different mutants may make use of different resources, and the relative resource concentrations change as the mutant frequencies change. It turns out that the mapping onto a linear system is still valid if resource abundances change due to external factors [24], but not if resources change in response to increasing or decreasing mutant frequencies *x*_*i*_. In this latter case, which corresponds to frequency-dependent selection, the conclusions drawn from quasispecies theory do not apply.

## Is quasispecies theory at odds with population genetics?

Several recent papers present quasispecies theory as a theory that is alternative to (and maybe even contradictory to) standard population genetics [6,7]. Is there any merit to this position? Is quasispecies theory somehow at odds with standard population genetics?

Let us investigate what form the quasispecies equations take in a simple example. Consider a single locus with two alleles a and A, and assume that the A allele has a selective advantage *s *over the a allele. Further, assume that allele a mutates into allele A, and likewise allele A into allele a, with probability *μ*. Then, in Eq. (1), we have *W*_AA _= (1 + *s*)(1 - *μ*), *W*_aA _= (1 + *s*)*μ*, *W*_Aa _= *μ*, *W*_aa _= 1 - *μ*, and hence (note that *x*_a_(*t*) = 1 - *x*_A_(*t*))



If we set the mutation rate *μ *to zero, then this equation turns into



that is, into the standard logistic equation that describes the rise of a beneficial allele in an otherwise homogeneous population. Thus, we can recover standard population dynamics from the quasispecies equations. Now, let us calculate the steady state solution of Eq. (4) for an arbitrary mutation rate. We set , and find



and of course *x*_a _= 1 - *x*_A_. For *μ *= 0, this expression becomes *x*_A _= 1, which simply means that the A allele will reach fixation in the absence of any mutation pressure. As *μ *increases, *x*_A _decreases, and *x*_a _increases. For a positive *μ*, even though the a allele is removed from the population by selection, it is constantly regenerated from the A allele by mutation pressure, and thus reaches a positive equilibrium frequency. If the mutation rate is sufficiently high, then the equilibrium frequency of the a allele, maintained by the balance of selection and mutation pressure, can be substantial. In summary, we find that for the case of a single locus with two alleles, the quasispecies model predicts logistic growth of the beneficial allele in the absence of mutations, and mutation-selection balance in the presence of mutations.

Now consider the multi-locus case. A classic paper on mutation-selection balance is the one by Kimura and Maruyama, written in 1966 [25]. In this paper, Kimura and Maruyama study the mutational load of a haploid, asexually reproducing population. I will now show that this model is also a special case of the quasispecies equations. Kimura and Maruyama assume that the frequency *x*_*i *_of a sequence with *i *mutations changes from one generation to the next according to (Eq. (3.1) in Ref. [25]):



where *w*_*i *_is the fitness of a sequence with *i *mutations, , and *μ *is the mutation rate (note that Kimura and Maruyama use *f*_*i *_instead of *x*_*i *_and 2*M *instead of *μ*). Now, define the mutation matrix *Q*_*ij *_as



and write the matrix *W*_*ij *_in Eq. (3) as *W*_*ij *_= *w*_*j*_*Q*_*ij*_. Then, we see that *E*(*t*) as defined in Eq. (2) becomes . Furthermore, the sum ∑_*j*_*W*_*ij*_*x*_*j *_in Eq. (3) runs from *j *= 0 to *j *= *i*, since *Q*_*ij *_= 0 for *i *<*j*. After introducing a new index *k *= *i *- *j*, we can rewrite the sum as



which demonstrates that Eq. (7) follows directly from the quasispecies equation Eq. (3). As a consequence, the quasispecies model is in agreement with the Haldane-Muller principle [26], which means that the mutational load *L* of a population described by the quasispecies model is in many (but not all) cases approximately given by *L*= 1 - *e*^-*μ*^. (Deviations from this principle arise for example from the presence of neutral mutations [27,28].)

Now that we have seen that quasispecies theory and mutation-selection balance are equivalent, the question remains whether Eigen just reinvented parts of population genetics, or actually contributed to the development of the field. While Eigen was not the first to consider mutation-selection balance (this concept goes back to Wright and Fisher in the early 20th century), by studying multi-locus mutation-selection equations at arbitrary mutation rate he was certainly at the forefront of theoretical population genetics in the late 1970s and early 1980s. The first analytic solutions to equations of the form Eq. (1) were found by Thompson and McBride in 1974 [29] and independently by Jones et al. in 1976 [30]. These works were directly influenced by Eigen's seminal paper of 1971 [10]. On the population genetics side, Moran was the first to solve Eq. (3) [31], also in 1976, but was unaware of the work by Eigen, Thompson, McBride, Jones, and coworkers.

**Figure 1:**
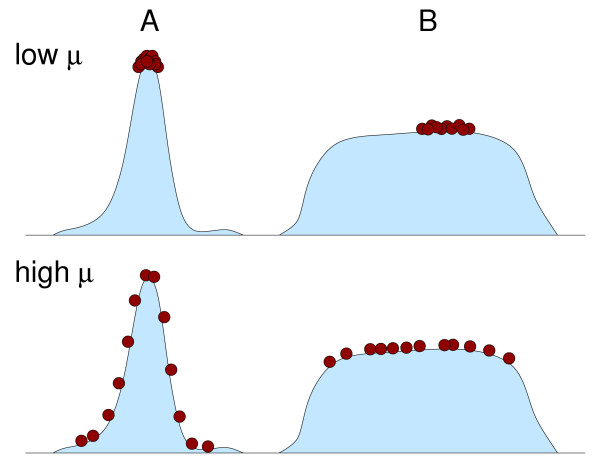
Schematic drawing of the survival-of-the-flattest effect. At low mutation rate *μ*, all individuals accumulate close to the top of the local fitness peak, and hence the individuals on peak A outcompete the individuals on peak B. At high mutation rate, most individuals on the steep peak A are located at low fitness values, while the individuals on the flat peak B remain close to the local optimum. As a consequence, the mean fitness of the individuals on peak B exceeds that of the individuals on peak A, and thus the former outcompete the latter.

One of the reasons why the quasispecies model is sometimes perceived to be at odds with standard population genetics is that it predicts (under certain conditions, I should add) that the equilibrium state of the population, which is given by the dominant eigenvector of the matrix *W*_*ij*_, is a stable mixture of closely related mutants. This mixture of mutants, also called a *mutant cloud *or *quasispecies*, does not necessarily have to contain the fastest-replicating individual sequence that exists in the fitness landscape. In other words, sequences with high fitness can lose out against sequences with lower fitness that have better support from their mutational neighbors [32,33], an effect which has been termed *survival of the flattest *(Figure 1).

It is important to understand that the emergence of a quasispecies is not something that has been put into the model ad hoc, but is a necessary consequence of the mutation-selection equations. We see in Eqs. (1) and (3) that the model is built on reproduction of individual sequences, but that mutations provide coupling between the different sequence types. When the mutation rate is low, then the quasispecies model predicts that the fastest-replicating sequence takes over the population, as we witness from the emergence of the logistic equation Eq. (5) at zero mutation rate. However, when the mutation rate is high, then the coupling between sequences caused by mutations can become stronger than the individual selection coefficients, and a quasispecies forms. Note that this effect will arise in any model of mutation-selection balance that correctly takes into account the coupling of different mutants at high mutation rate.

## Does quasispecies theory apply to finite populations?

In the previous section, I have established that the quasispecies model is equivalent to the theory of mutation-selection balance in an infinite, haploid, asexual population. However, this equivalence does not necessarily imply that the quasispecies model applies to populations of RNA viruses, because these populations are finite. Jenkins et al. [5] argue that the total sequence space of an RNA virus is much larger than the sequence space a finite population of realistic size can cover, and that therefore the deterministic equations of the quasispecies model are inapplicable, because virus evolution is dominated by random genetic drift. A priori, this is a reasonable objection, and we have to test whether the quasispecies equations are indeed useless in any realistic setting, or whether maybe complete coverage of the sequence space is not necessary to observe quasispecies effects. (By *quasispecies effects*, I mean that the population behaves in a way that can only be explained through strong mutational coupling between genotypes. An example would be the observation of the survival of the flattest effect.)

First, let us have a look at some theoretical studies of finite populations that have been carried out within the context of the quasispecies model [19,34-38]. In general, in these studies the deterministic equations are taken as the starting point, and then the authors derive corrections to these equations that take into account deviations from the deterministic behavior caused by the finite population size. Thus, at least in these model systems, the deterministic equations provide a reasonable starting point to understand the population dynamics. Van Nimwegen et al. make this point particularly clear by showing that in certain cases, we can understand the behavior of a finite population from a deterministic description of a population that occupies the sequence space around a local optimum [37]. In this model, information about other local optima (which would be available to an infinite population) is not necessary to accurately describe the behavior of the finite population on the given local optimum.

However, one could still object that these models may be describing idealized and simplified situations that differ too much from the reality of RNA viruses to be of any relevance. To counter this argument, we have to ask whether there is a more general way to determine the relevance of quasispecies theory to finite populations of RNA viruses. The hallmark of quasispecies theory (and of course of any theory of mutation-selection balance) is that for a sufficiently high mutation rate, we must take into account the formation of a quasispecies to obtain a faithful description of the population dynamics. Therefore, the question is under what conditions does mutational coupling become so strong that we can observe quasispecies effects in a finite population. Can theory help us to address this question?

In the formation of a quasispecies, the population minimizes the mutational load by accumulating sequences that have a reduced probability to suffer from deleterious mutations [27,28,39,40]. This effect has been termed *evolution of mutational robustness *[27]. Van Nimwegen et al. studied this effect for RNA evolution, and found that mutational robustness evolved as long as the product of mutation rate *μ *and effective population size *N*_*e *_was significantly larger than one, *μN*_*e *_≫ 1 [27]. I have recently made similar observations in simulations of protein evolution [41]. What is interesting about the latter simulations is that in the regime of mutational robustness, the proteins continued to accumulate mutations, and in fact accumulated mutations faster than in the regime in which mutational robustness did not evolve. This observation demonstrates that the existence of a stable master sequence is not a necessary consequence of quasispecies evolution, in contrast to the key assumption of the study by Jenkins et al. [5]. These results can be understood with the theory of quasispecies fixation, which shows that an individual invading sequence has a positive fixation probability precisely when the mutant cloud that this sequence will spawn has higher fitness than the currently established mutant cloud, regardless of the individual fitness of the invading sequence [42,43]. Note that, in line with the previous section, this theory is again equivalent to the general theory of fixation in a haploid, asexually replicating population [44,45].

Finally, the recent paper by Comas et al. [7] also provides evidence that quasispecies effects can be observed in surprisingly small populations, populations far too small to cover the relevant sequence space. Comas et al. studied to what extent the survival-of-the-flattest effect would be affected by population size, and found that population size played only a minor role in determining the position of the critical mutation rate at which the flatter strain began to outcompete the fitter strain. (I had previously found similar results in simulated RNA evolution [46].) Even in populations of size 250, Comas et al. consistently observed outcompetition of the fitter strain by the flatter strain at high mutation rate. Note that the digital organisms in these experiments had sizes of between 54 and 272 instructions, chosen from an alphabet of 28 different instructions, so that the complete sequence space of these organisms was between 10^47 ^and 10^68 ^sequences large. Clearly, a population of size 250 (or even several thousand, for that matter) cannot even come close to complete coverage of such a huge sequence space.

The previous paragraphs show that on purely theoretical grounds, there is no reason to assume that quasispecies effects cannot play a role in finite populations of RNA viruses. Nevertheless, to date we have no experimental evidence that unequivocally demonstrates such effects in a specific experimental system. On the other hand, selection for specific, individual mutants is common (see e.g. Ref. [47]). What does this experimental evidence imply for quasispecies theory? First, quasispecies theory covers both cases, those in which mutational coupling can be neglected, and those in which it can't. The latter is a second-order effect that becomes relevant only when there is no strong selection for individual sequences [40]. Thus, it is not surprising that in cases where selection is strong, such as in the case of resistance or escape mutants, we don't see quasispecies effects. Second, because quasispecies effects are second-order, it may be difficult to detect them experimentally, and experiments more sensitive than the ones carried out to date may be necessary to demonstrate their presence unequivocally.

In summary, we currently have no evidence (theoretical or experimental) that contradicts the existence of quasispecies effects in finite populations of RNA viruses, but we also have no experimental evidence in favor of it. Theoretical studies and computer simulations indicate that quasispecies effects should become important when the product of effective population size and genomic mutation rate is significantly larger than one. Since for many RNA viruses the genomic mutation rate is already on the order of one [48,49], even moderately large populations of RNA viruses, or populations that undergo regular bottlenecks, are candidates for quasispecies behavior.

## Does quasispecies theory predict an error threshold?

The error threshold is probably one of the most misunderstood concepts of quasispecies theory. Eigen described the error threshold in his 1971 paper as a limit to the amount of information a genome can store at a given mutation rate [10]. If the mutation rate is increased beyond this limit, then the population structure breaks down, and the population disperses over sequence space.

**Figure 2:**
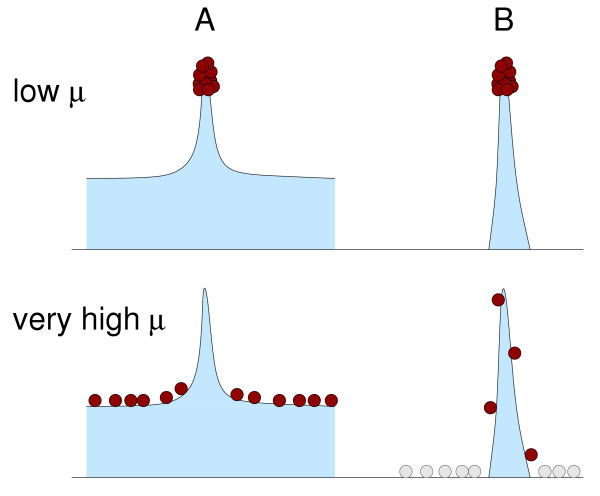
Schematic drawing of the error threshold. If a fitness landscape has a positive minimum fitness (case A), then at a sufficiently high mutation rate all individuals are pushed to this minimum level. The selective strength on the narrow peak is not sufficient to counteract the mutation pressure. If a fitness landscape has no minimum fitness (case B), then the mutation pressure pushes a large fraction of the population to zero fitness. The individuals with zero fitness (shown in gray) are inviable, and thus do not compete with the individuals on the fitness peak. Therefore, a few individuals will always remain on the top of the fitness peak. Note that this conclusion holds only when two assumptions are met: (i) The population is infinite. (Otherwise, stochastic effects push the population away from the peak, and we observe Muller's ratchet.) (ii) Selection is soft, that is, only relative fitness differences matter, and the overall population size is held constant at all times. (If selection is hard, then the population size will decline as the mutation rate is increased, and eventually the population can go extinct. This case is mutational meltdown.)

The first important point to understand about the error threshold is that it is a deterministic effect. This means that the position of the error threshold depends only weakly on the population size, and that even in an infinite population the error threshold occurs at a finite mutation rate [19,35]. In this way, the error threshold differs markedly from Muller's ratchet [50], which occurs only at finite population sizes and disappears in the deterministic limit. Second, the error threshold's existence and position depend on the choice of the fitness landscape [51-56]. Even though the error threshold is usually perceived as a general prediction of quasispecies theory, most of the literature that studies the error threshold considers only the single-peak fitness landscape, and disregards all other possible fitness functions [10,15-21]. The single-peak fitness landscape has the unrealistic property that all sequences have a positive replication rate, that is, there are no lethal mutants. As a result, at high mutation rates these sequences compete with the master sequence (the single sequence that has higher fitness than all other sequences), and can win this competition at sufficiently high mutation rate by sheer abundance. Can the error threshold occur in a more realistic fitness landscape that contains lethal genotypes? No. Wagner and Krall have proven mathematically that the condition for the existence of an error threshold is precisely the complete absense of lethal genotypes [53]. An intuitive explanation for this result is provided in Fig. 2.

There is certainly no lack of lethal mutants in viruses [57,58], and as a consequence, viruses cannot suffer from an error threshold as defined by Eigen. If this is the case, then how can we understand the concept of lethal mutagenesis, which has recently proven successful in a variety of viruses [59-62], and for which the error threshold is generally cited as the underlying theory? The truth of the matter is that the two concepts are mostly unrelated. To understand the difference between the two, we have to understand the difference between soft and hard selection. Soft selection means that the population size is held constant, regardless of the mean fitness of the population. Under soft selection, populations cannot go extinct by definition. Since the quasispecies model is usually studied in the context of soft selection (even though it can be generalized to hard selection), the error threshold *per se *makes no statements about population extinction. The alternative model is hard selection, where the population size will decline if the mean fitness of the population is too low. Extinction due to mutation pressure can occur under hard selection, and is usually called mutational meltdown [63-65]. Mutational meltdown will operate in any fitness landscape, as long as the population size is sufficiently small, the mutation rate sufficiently large, or the hard selection pressure sufficiently strong. While lethal mutagenesis is probably a valid antiviral therapy, referring to it as an error-threshold related effect is at best a misnomer, and can at worst lead to poor treatment decisions brought about by a misunderstanding of the actual evolutionary dynamics that unfold under lethal mutagenesis.

Finally, it is interesting to note that in certain fitness landscapes, several error-threshold-like transitions can occur one after the other as the mutation rate increases [66]. At each transition, the population loses the ability to take advantage of a particular region of sequence space, and delocalizes over this region, while remaining localized in other regions. Tannenbaum and Shakhnovich have termed this effect the *error cascade *[66], and in fact, the survival-of-the-flattest effect [32,33,46] can be considered as a special case of this error cascade.

## Conclusion

To summarize, I have provided arguments for the following conclusions: Quasispecies theory is in perfect agreement with population genetics, it can make usefull predictions for finite populations if the product of population size and mutation rate is large, and it predicts an error threshold only for fitness landscapes that lack lethal mutants, and which therefore have little relevance for virus evolution.

However, these arguments do not imply that quasispecies theory is the final answer to all questions of virus evolution. Quasispecies theory has its short-comings that need to be addressed in future modeling work. Ironically, the biggest shortcoming of quasispecies theory, as far as I can see, does not have its origin in quasispecies theory being at odds with population genetics, but rather in quasispecies theory being too similar to the population genetics theory of asexual, haploid organisms. Viruses differ from other forms of life in that they don't have a well-defined ploidy. When a single virus particle infects a cell, the virus can be considered a haploid organism, and indeed the quasispecies model makes this assumption. However, frequently several virus particles coinfect the same cell, in which case the ploidy is given by the number of coinfecting particles. Standard population genetics has no model for such a variable-ploidy organism, and only a handful of authors have considered the theoretical implications of viral coinfection in detail [67-81].

## Authors' contributions

COW carried out all aspects of this work.
